# Long-term health-related quality of life in survivors of sepsis: an epidemiological study

**DOI:** 10.1186/cc14098

**Published:** 2015-03-16

**Authors:** CE Battle, G Davies, M Vijayakumar, PA Evans

**Affiliations:** 1 NISCHR HBRU Morriston Hospital, Swansea, UK; 2 College of Medicine, Swansea University, Swansea, UK; 3 Ed Major Critical Care Unit, Morriston Hospital, Swansea, Swansea, UK

## Introduction

Survivors of sepsis report persistent problems that can last years after hospital discharge. The main aim of this study was to investigate long-term health-related quality of life in survivors of SIRS and sepsis compared with Welsh normative data, controlling for age, length of stay and pre-existing conditions. The second aim was to investigate any differences in long-term health-related quality of life specifically with the patients categorised into three groups: SIRS, uncomplicated sepsis, and severe sepsis/septic shock.

## Methods

A prospective study design was used in order to investigate all sepsis patients either presenting to the emergency department or admitted to the ICU of a regional trauma centre. A total of 106 patients were recruited and all patients were considered eligible as per the SIRS and sepsis criteria [1]. The Sepsis-related Organ Failure Assessment score was determined over the first 24 hours to assess organ function. Patients were assigned to groups as follows: sterile SIRS; uncomplicated sepsis; severe sepsis or septic shock as per the criteria. Assignment into groups was blinded and performed by an intensive care specialist independent of the study. Baseline demographics, clinical characteristics and outcomes were collected and surviving patients were sent a SF-12v2 survey at between 6 months and 2 years post hospital discharge.

## Results

A total of 106 patients were included in the study. A mortality rate of 34% was recorded, leading to a final response rate of 72% by the end of the data collection period. Quality of life was significantly reduced in all patients when compared with local normative data (all *P *< 0.0001). Reductions in the physical components of health-related quality of life were more pronounced in severe sepsis/septic shock patients when compared with uncomplicated sepsis and SIRS patients.

## Conclusion

This is the first observational study to specifically focus on the different groups of SIRS and sepsis patients to assess long-term quality of life. Local population norms were used for comparison, rather than wide geographical norms that fail to reflect the intricacies of a country's population. Significant reductions in quality of life were found in severe sepsis/septic shock patients compared with in uncomplicated sepsis and SIRS patients, when controlling for age, preexisting conditions, hospital and ICU length of stay.
